# The Relationship between Electrocardiographic Changes and Prognostic Factors in Severely Symptomatic Pulmonary Hypertension

**Published:** 2019-01

**Authors:** Seyed Reza Seyyedi, Babak Sharif-Kashani, Makan Sadr, Mandana Chitsazan, Majid Malekmohammad, Atefeh Abedini, Fateme Monjazebi, Farah Naghashzadeh

**Affiliations:** 1 Lung Transplantation Research Center, Department of Cardiology, National Research Institute of Tuberculosis and Lung Diseases (NRITLD), Shahid Beheshti University of Medical Sciences, Tehran, Iran; 2 Tobacco Prevention and Control Research Center, NRITLD, Shahid Beheshti University of Medical Sciences, Tehran, Iran; 3 Virology Research Center, NRITLD, Shahid Beheshti University of Medical Sciences, Tehran, Iran; 4 Tracheal Diseases Research Center, NRITLD, Shahid Beheshti University of Medical Sciences, Tehran, Iran; 5 Chronic Respiratory Diseases Research Center, NRITLD, Shahid Beheshti University of Medical Sciences, Tehran, Iran.

**Keywords:** Electrocardiography, Pulmonary hypertension, Prognosis, Pulmonary

## Abstract

**Background:**

The prognostic role of the electrocardiogram (ECG) in PH is not fully known. We aimed to evaluate ECG abnormalities in severe PH, the association of ECG patterns with known prognostic factors and to determine whether ECG abnormalities were associated with decreased survival in patients with severe PH.

**Materials and Methods:**

Fifty-two patients with severe PH were included. Clinical assessment included basic demographics, complete physical examination, determination of WHO FC, measurement of N-terminal pro-BNP, 12-lead electrocardiography, transthoracic echocardiography, right heart catheterization (RHC) and six minute walk test (6MWT).

**Results:**

Heart rate was correlated with NT-proBNP (r=0.54; p-value: 0.0001) and was higher in patients with severe RV dysfunction (93±12 vs. 83±4 bpm in moderate RV dysfunction). P-pulmonale was present in 51.9% of the patients and was significantly associated with severe RV dysfunction. qR in V1 (48.1%) was significantly associated with 6MWT and severe RV dysfunction. Overall, 10 patients died. Based on Kaplan-Meier results, median survival time was 38 months and estimated survival at 1 year, 3 years, and 5 years was 88%, 80% and 71 % respectively. In Cox regression analysis WHO FC, 6MWT, pericardial effusion, NT-pro BNP, heart rate, ST depression in V1 to V3, and presence of qR in V1 were predictors of mortality. After controlling for covariates, only NT-proBNP was independently associated with decreased survival.

**Conclusion:**

ECG changes including P-pulmonale, qR pattern in V1, and heart rate indicative of right ventricular dysfunction are associated with prognostic factors in severe PH and may be a useful tool in the follow-up.

## INTRODUCTION

Pulmonary hypertension (PH) is defined as mean pulmonary artery pressure 25 mmHg or more at rest, measured by right heart catheterization (1). PH consists of a group of disorders characterized by progressive thickening of pulmonary vasculature, leading to right ventricular dysfunction and death (2). PH is more common in females (3) and its true prevalence is unclear, probably due to broad range of etiologies. However, the estimated prevalence in adults is 6 to 15 cases per one million in different registries (4,5). Despite recent advances in the treatment of patients with PH, the prognosis in many patients remains poor (6). The estimated mortality rate for PH was 6.5 per 100,000 in the United States in 2010 (7). The World Health Organization functional class (WHO FC), the 6-minute walking distance (6MWD), N-terminal-pro B-type natriuretic peptide (NT-proBNP) and Right Ventricular (RV) dysfunction are known predictors of prognosis in PH (8–12).

Electrocardiogram is a simple, inexpensive, and non-invasive test. However, its prognostic role in patients with PH remains uncertain. Common ECG patterns seen in PH include right atrial abnormalities, right axis deviation, right ventricular hypertrophy with strain pattern (13). In a study by Bossone et al in 2002, the presence of P-pulmonale and qR in V1 were independent predictors of increased mortality in patients with primary PH (14). In another study, the presence of qR in V1 was shown to be a sign of advanced pulmonary arterial hypertension and was a significant prognostic factor (15). In a recent study, right axis deviation (RAD) of QRS complex was present in 23% of patients with PH and was associated with a high positive predictive value (92–93%) for PH (16). Therefore, in this study we aimed to evaluate the distribution of ECG abnormalities in our severe PH patients and determine the association of ECG patterns with established prognostic factors and mortality in patients with severe PH.

## MATERIALS AND METHODS

This is a prospective, observational study conducted in the PH Clinic, Department of Cardiovascular Diseases at Masih Daneshvari Hospital, a pulmonary tertiary center, Tehran, Iran, between March 2012 and January 2017. The hospital runs a specific PH Clinic, visiting referred PH patients from all across the country for diagnosis and/or treatment of PH.

All patients referred to the clinic are evaluated comprehensively for suspected PH. Diagnostic work-ups follows recommendations of current international guidelines (17). All consecutive patients with established diagnosis of PH were eligible to be included in the study if they were classified as severe PH. Patients with PH due to left heart diseases, lung diseases and/or hypoxia were excluded. After a diagnosis of PH was made, treatment strategies were evaluated and applied by expert cardiologists and pulmonologists, according to the guidelines (17).

Clinical assessment included basic demographics, complete physical examination, determination of WHO FC, measurement of N-terminal pro-BNP (NT-proBNP), 12-lead electrocardiography, transthoracic echocardiography, right heart catheterization (RHC) and six minute walk test (6MWT). All data were collected during single hospitalization. The study protocol was approved by Institutional Ethics committee. After completely explaining the study protocol to the patients, written informed consent was obtained from all participants.

A standard12-lead ECG (10 mm=1 mV, 25 mm/s) was performed by trained technicians in a supine position in all patients. ECGs were analyzed manually using magnifying lens by two cardiologists. They were blinded to the patients’ data and symptoms. Any uncertainty was resolved by consensus. Assessed ECG parameters included: cardiac rate and rhythm, P wave amplitude and duration, PR interval duration, QRS morphology and duration, QRS electrical axis, presence of conduction abnormalities, ST-segment and T wave morphologies. P-pulmonale as an indicative of right atrial enlargement was defined as upright P wave in lead II >2.5 mm (18). Right axis deviation (RAD) was considered as axis of >90° (19).Complete RBBB was defined according to AHA/ACCF/HRS Recommendations: QRS duration ≥120 ms, rsr′, rsR′, or rSR′ in leads V1 or V2 (The R′ or r′ deflection is usually wider than the initial R wave) or a wide and notched R wave in V1 and/or V2. S wave of greater duration than R wave or > 40 ms in leads I and V6, Normal R peak time in leads V5 and V6 but >50 ms in lead V1 (19). Incomplete RBBB was defined by QRS duration between 110 and 120 ms. Other criteria are the same as for complete RBBB (19). ECG criteria of RVH was defined as presence of qR in V1, S>R in leads I, II, III (S1S2S3), and S1Q3 pattern (20).

Transthoracic Echocardiography (TTE) was performed in all patients with Vivid 7 Dimension echocardiography machine (GE Healthcare, Horten, Norway) with a 4 MHz probe. Systolic right ventricular dysfunction was diagnosed in the presence of moderately to severely depressed right ventricular free wall kinesis. Six minute walk test (6MWT) was performed according to the American Thoracic Society statement (21). RHC was performed in a supine position from the right femoral vein using a swan-Ganz catheter. Mean pulmonary arterial pressure (mPAP) was measured in all patients during RHC. All patients were evaluated at least every 3 months or sooner in the case of clinical deterioration. For the purpose of this study, follow-ups ended on 31th January 2017. All-cause mortality was confirmed by reviewing the death certificate or medical records.

### Statistical analysis

Mann-Whitney U test was used to compare NT-proBNP and 6MWT in the presence of ECG parameters and other qualitative variables. Chi-squared or Fisher exact tests were used for comparing qualitative variables. Spearman correlation was used for testing correlation between heart rate and NT-proBNP and 6MWT. Survival times are described using Kaplan-Meier survival estimates. Variables were tested as possible predictors of mortality using Cox proportional hazard model and Hazard ratios (HRs) and 95% confidence intervals (CIs) are reported. First, each variable was entered in the model, individually. Then, variables with p-value more than 0.2 entered multivariate analysis. The data were analyzed using SPSS software version 18 (Chicago, IL, USA). All reported p-values are two-tailed, and p-values of less than 0.05 were considered statistically significant.

## RESULTS

A total of 52 patients, including 10 (19.2%) males and 42 (80.8%) females enrolled to the study. The mean age was 41.46±15.22 years. The majority of study population comprised of patients with the diagnoses of idiopathic pulmonary hypertension (IPAH; n=28, 53.8%), Eisenmenger’s syndrome (n=11, 21.2%) and chronic thromboembolic pulmonary hypertension (CTEPH; n=9, 17.3%). The etiology of pulmonary hypertension in the remaining 4 patients was PH associated with connective tissue disease (n=2, 3.8%), pulmonary veno-occlusive disease (PVOD; n=1, 1.9%) and thalassemia (n=1, 1.9%). All patients were in World health organization (WHO) function classes III (n=44, 84.6%) and IV (n=8, 15.4%). Clinical characteristic of the study population are showed in Table 1.

**Table 1.** Basic characteristics

**Variable**		**Mean±SD or N(%)**
**WHO Functional Class**	III	44 (84.6)
	IV	8 (15.4)
**Etiology of pulmonary hypertension**	IPAH	28 (53.8)
	CTEPH	9 (17.3)
	Eisenmenger’s syndrome	11 (21.2)
	Connective tissue disorder	2 (3.8)
	PVOD	1 (1.9)
	Thalassemia	1 (1.9)
mPAP mmHg^*^		80 (71.25–95)
**Right ventricular dysfunction, n**	Moderate	8 (15.4)
	Severe	44 (84.6)
6MWT, meter^*^		240 (180–288.25)
NT-proBNP, pg/ml^*^		1850 (825–2499.50)
**Heart rate, bpm**		92±12
**Rhythm**	Sinus	48 (92.3)
	Atrial fibrillation	3 (5.8)
	Atrial flutter	1 (1.9)
**P-pulmonale**		27 (51.9)
**QRS Axis**	Normal axis	15 (28.8)
	RAD	37 (71.2)
**ST depression in II, III, aVF**		28 (53.8)
**ST depression in V_1_–V_3_**		41 (78.8)
**ST depression in V_4_–V_6_**		29 (55.8)
**Complete RBBB**		19 (36.5)
**Incomplete RBBB**		23 (44.2)
**qR in V_1_**		25 (48.1)
**S1S2S3**		3 (5.8)
**S1Q3**		10(19.2)

* displayed as median (Interquartile range)

SD: Standard Deviation; N: Number; IPAH: Idiopathic Pulmonary Arterial Hypertension; CTEPH: Chronic Thromboembolic Pulmonary Hypertension; PVOD: Pulmonary Veno-Occlusive Disease; mPAP: mean Pulmonary Artery Pressure; 6MWT: 6 minute walk test; NT-proBNP: N-terminal-proBNP; RAD: Right Axis Deviation; RBBB: Right Bundle Branch Block

Twenty-nine patients (55.8%) were receiving combination therapy with oral Tadalafil and oral Bosentan. Intravenous Iloprost was administered in 23 patients (44.2%). Tadalafil was discontinued in six patients due to intolerance (severe headaches). In five patients, Bosentan caused increased hepatic aminotransferases and therefore, it was discontinued. The patients in moderate and severe RV dysfunction groups were comparable regarding treatment regimen (p-value=0.45). Sinus rhythm was present in 48 (92.3%) patients. Three patients (5.8%) and one patient (1.9%) had atrial fibrillation and atrial flutter, respectively. The mean heart rate was 83±4 bpm in patients with moderate RV dysfunction and 93±12 bpm in patients with severe RV dysfunction. The observed difference was statistically significant (P=0.03). There was a significant correlation between heart rate and NT-proBNP level (correlation coefficient, 0.54; p-value: 0.0001). Heart rate was inversely correlated with 6MWT (correlation coefficient, −0.43: p-value: 0.001). P-pulmonale was more common in patients with severe RV dysfunction, compared to those with moderate RV dysfunction (P=0.02). There were no relationships between presence of P-pulmonale and NT-proBNP levels and distance walked in 6MWT (p-values of 0.144 and 0.56, respectively). Right axis deviation of QRS was not associated with NT-proBNP, 6MWT and RV dysfunction.

ST-segment depression in precordial and inferior leads, complete and incomplete RBBB were not associated with markers of poor prognosis. qR pattern in V1 was significantly associated with decreased performance in 6MWT (216.2±67.2 m compared to 271.3±97.2 m, P= 0.03). Presence of qR was also associated with higher NT-proBNP levels (1992±856 versus 1220±863, p-value=0.002) and severe RV dysfunction (p-value=0.004). S1S2S3 and S1Q3 patterns were not significantly associated with prognostic factors.

**Figure 1.**
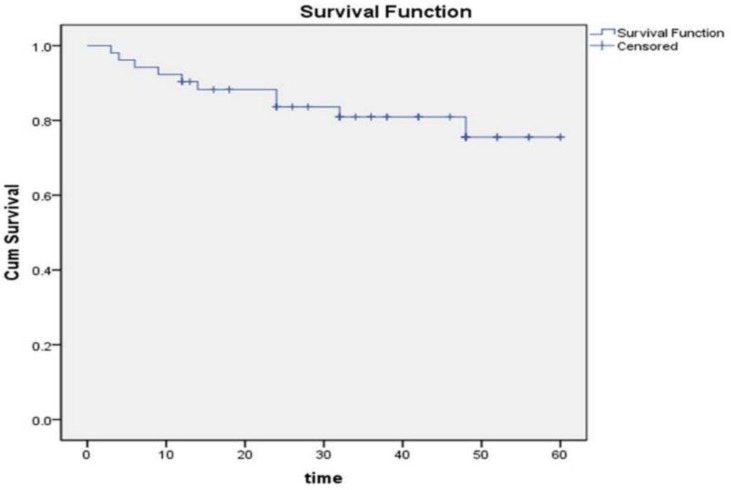
Kaplan-Meyer survival Curve of patients with pulmo

The median follow-up was 33 months (IQR: 18–48). There were no lost to follow-up cases. Overall ten patients died. The cause of death was progressive right heart failure in all cases. For the remaining 42 patients, survival times were censored at the time of last visit. Median survival time was 38 months (Figure 1). The estimated proportion of patients surviving at 1 year, 3 years, and 5 years were 88% (95%CI, 78 to 98%), and 80% (95% CI, 68% to 92%), and 71% (95% CI, 51% to 91%) respectively.

In univariate Cox PH model, WHO FC, 6MWT, pericardial effusion, NT-pro BNP, heart rate, ST depression in V1 to V3, and presence of qR in V1 were predictors of mortality. However, in multivariate model after adjusting for covariates, only NT-proBNP was independent predictor of mortality (Adjusted Hazard ratio: 1.003, 95% CI 1.001 to 1.004) (Table 2).

**Table 2.** Univariate and multivariate cox proportional hazard model

		**Univariate analysis**			**Multivariate analysis**		
**variable**		HR	95% CI	P value	HR	95% CI	P value
**Age**		1.0	0.95–1.04	0.98			
**Sex**	**Male**	1	0.23–14.94	0.55			
	**Female**	1.88					
**WHO FC**	**III**	1	2.07–24.99	0.002			
	**IV**	7.2					
**6MWT**		0.988	0.977–0.998	0.024			
**mPAP**		1.03	0.99–1.05	0.106			
**RV dysfunction**	**Moderate**	1	0.26–16.95	0.478			
	**Severe**	2.12					
**Pericardial effusion**		5.72	1.61–20.24	0.007			
**NT-pro BNP**		1.002	1.001–1.004	0.005	1.003	1.001–1.004	0.002
**Uric acid**		1.28	0.93–1.77	0.127			
**Heart rate**		1.072	1.027–1.118	0.001			
**P-pulmonale**		1.67	0.46–6.03	0.43			
**QRS axis**	**Normal axis**	1	0.44–10.10	0.34			
	**RAD**	2.12					
**ST depression in II, III, aVF**		0.96	0.27–3.33	0.95			
**ST depression in V_1_–V_3_**		3.92	1.12–13.66	0.032			
**ST depression in V_4_–V_6_**		1.92	0.49–7.45	0.34			
**Complete RBBB**		1.28	0.36–4.54	0.70			
**Incomplete RBBB**		0.30	0.06–1.45	0.13			
**qR in V_1_**		9.54	1.20–75.50	0.032			
**S1S2S3/S1Q3**		2.30	0.28–18.51	0.431			

## DISCUSSION

In the present study, P-pulmonale, heart rate, and qR in V1 were associated with severe RV dysfunction; heart rate and qR in V1 were significantly associated with 6MWD; heart rate was correlated with NT-proBNP levels; WHO FC, 6MWD, presence of pericardial effusion on echocardiography, NT-proBNP level, heart rate, ST segment depression in V1–V3, and qR pattern in V1 were significantly associated with mortality; while only NT-proBNP was independent predictor of mortality.

In pulmonary hypertension, increased resistance in pulmonary vasculature leads to increased RV afterload. With progression of the disease, RV hypertrophy and dilation ensues and RV failure is the ultimate result of progressive RV dysfunction. The presence of qR in lead V1 is reflective of right ventricular enlargement. qR pattern in V1 was present in almost half of our patients (51.9%) and it was associated with worse exercise capacity and RV function and higher NT-proBNP. The association of qR in V1 with RV dysfunction in PH subtypes was also shown in previous studies. Nagai et al. in a study of 31 patients with PH showed that the presence of qR in V1 was an independent determinant of RV dysfunction (22). In a study by Waligora et al. qR in V1 was present in 43.4% of the PH patients and was significantly associated with RV dysfunction and decreased survival (15).

RV dysfunction also leads to decreased stroke volume, and increased neuro-hormonal stimulation of the myocardium probably explains increased heart rate in PH patients (14). Bossone et al. showed that increased heart rate increases the risk of death (14). In our study increased heart rate was associated with higher NT-proBNP, poorer performance in 6MWT, severe RV dysfunction and death. However, after adjustment for possible cofounders, heart rate did not remain an independent predictor of mortality.

In our analyses, Class IV WHO FC and lower performance in 6MWT were significantly associated with higher mortality. It was in line with previous studies in which WHO FC and 6MWD were independent predictors of survival (9,23, 24). Moreover, our analyses showed higher mortality in patients with pericardial effusion on echocardiography. Previous investigators reported that pericardial effusion on echocardiography reflects the severity of right heart failure and predicts adverse outcomes in patients with primary pulmonary hypertension (25). The US Registry to Evaluate Early and Long-Term PAH Disease Management (REVEAL), evaluating 2716 patients with PAH, identified the etiology of PAH, WHO FC, 6MWD, and pericardial effusion on echocardiogram as predictors of mortality (9).

There are several limitations to this study. We did not re-evaluate ECG changes during and at the end of the follow-up period in most of the patients. So, future studies with larger sample size and monitoring of the prognostic markers at intervals can better reveal correlation of ECG parameters with disease severity and progression.

## CONCLUSION

Electrocardiographic changes including P-pulmonale, qR pattern in V1, and heart rate were associated with RV dysfunction and prognostic markers in patients with severe pulmonary hypertension.
